# Cerebral Subdural Hematoma Following Spinal Anesthesia: Report of Two Cases

**DOI:** 10.1155/2012/352028

**Published:** 2012-03-27

**Authors:** Mehrdad Moradi, Shoaleh Shami, Fariba Farhadifar, Karim Nesseri

**Affiliations:** ^1^Department of Neurosurgery, Faculty of Medicine, Kurdistan University of Medical Sciences, Sanandaj, Iran; ^2^Faculty of Nursing and Midwifery, Kurdistan University of Medical Sciences, Sanandaj, Iran; ^3^Department of Gynecology and Obstetrics, Faculty of Medicine, Kurdistan University of Medical Sciences, Sanandaj, Iran; ^4^Department of Anesthesia, Faculty of Medicine, Kurdistan University of Medical Sciences, Sanandaj, Iran

## Abstract

Postdural puncture headache and cerebral subdural hematoma are among complications of spinal anesthesia with some common characteristics; however misdiagnosis of these two could result in a catastrophic outcome or prevent unwanted results by urgent interventions. With the purpose of increasing awareness of such complications and a speedy diagnosis, we report two cases of postspinal anesthesia headache that was timely diagnosed as cerebral subdural hematoma and prevented the likelihood of a disastrous outcome.

## 1. Introduction

Spinal anesthesia is an ordinary and daily practice of anesthesiologists. Although the incidence of benign complications is relatively high, only 0.05% of complications are critical [1, 2]. Cerebral subdural hematoma (SDH) is among these rare, serious, and life-threatening complications, that may be misdiagnosed with postdural puncture headache (PDPH) [3, 4]. Because of some common characteristics of typical PDPH with cerebral subdural hematoma (SDH), awareness of clinicians regarding its signs and symptoms could result in the prevention of unwanted outcomes through urgent intervention. This leads us to present two patients of cerebral SDH following spinal anesthesia.

**Case 1**

A 27-year-old woman, ASA I, with history of previous cesarean section under spinal anesthesia 29 months ago was admitted at 37 weeks gestational age of her second pregnancy with uterine contractions and underwent emergency cesarean section at night. Routine preoperative exams including coagulation tests were within the normal limits and she had no signs of preeclampsia, history of head trauma, headache, or connective tissue disorders during her pregnancy. Spinal anesthesia was performed in sitting position with midline approach at L3-L4 space, using a Whitacre 25-gauge needle. The drugs used included 2 mL of 0.5% hyperbaric bupivacaine (10 mg) and 5 *μ*g of sufentanil and were administered successfully on the first attempt.

She was hydrated with 1500 mL of 0.9% normal saline intravenously prior to surgery and an additional 1500 mL of 0.9% normal saline was administered during surgery. During anesthesia systolic blood pressure was decreased by 30% and 2 bolus doses of ephedrine (total of 10 mg) were administered. She showed signs of sinus bradycardia and nausea and was treated with 0.5 mg atropine too.

At the end of surgery, she complained of shivering and headache and was treated with 35 mg of meperidine which was effective and the headache subsided. In the ward the patient complained of headache once again and PDPH was diagnosed and treated with intravenous morphine, hydration, and bed rest. She ambulated 24 hours later and was discharged from hospital with partial relief of symptoms after 48 hours. Thirteen days after the puncture, the patient remained with constant, nonpostural headache, and without neurological changes.

**Figure 1:**
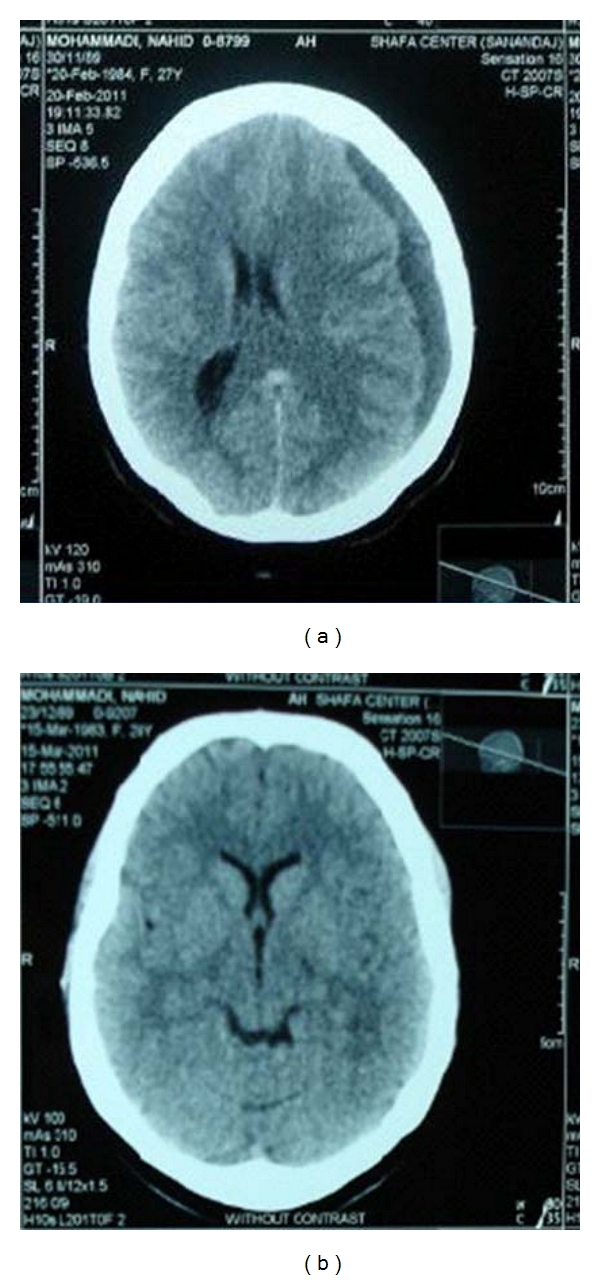
(a) Brain computed tomography scan showed a late subacute SDH of the left frontotemporal region and midline shift. (b) Normal control Computed tomography scan.

During this period, she was in supine position mostly and went to her surgeon twice and was advised to continue taking the oral analgesics, but the pain did not improve. After 13 days, nausea, vomiting, and right side hemiparesis were added to her unbearable headaches. She was referred to a neurologist and brain computed tomography (CT) scan was ordered which showed a late subacute cerebral SDH on the left side (Figure 1). Subsequently, because of neurological symptoms and a large size lesion, she underwent surgery and the hematoma lesion was removed. Her symptoms resolved entirely and the patient did not develop any further symptoms. The followup of the patient continued for three months and control CT scan was normal.

**Case 2**

A 49-year-old woman was referred to our hospital for severe, nonpostural headache as a result of undergoing umbilical herniorrhaphy under spinal anesthesia two days before. Based on medical records received, she had no history of medical disease and head trauma before and after surgery and had not taken any drugs before surgery. Preoperative hematologic laboratory values were normal. She had been hydrated with 500 mL of 0.9% normal saline before the procedure, and dural puncture had been performed at the L3-L4 interspace in the sitting position, using a midline approach with Whitacre 25-gauge spinal needles, using 15 mg of 0.5% hyperbaric bupivacaine.

**Figure 2:**
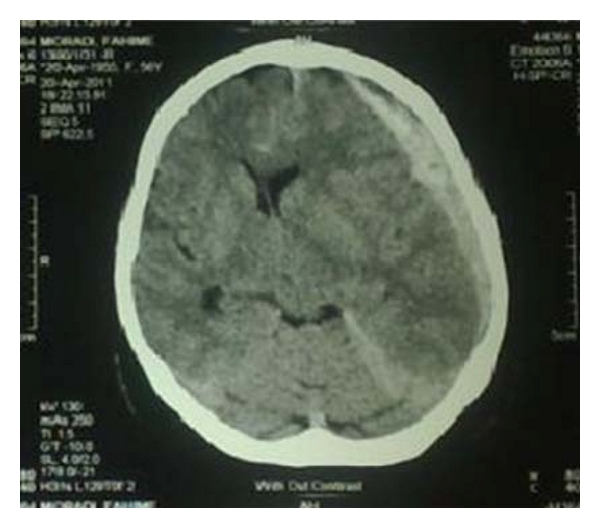
Brain CT scan showed acute SDH of the left frontotemporal region and midline shift.

Headaches started immediately after the patient was discharged from the postoperative care unit and diagnosed as PDPH. The patient had been treated with morphine sulfate, hydration, and bed rest. She was in supine position and restricted to ambulation because of her headache. Twenty-four hours later, the patient had still not responded to treatment and left eye ptosis, right hemiparesis, nausea, and vomiting were also added to the symptoms. Patient had undergone a CT scan (Figure 2) which detected acute subdural hematoma, and the patient was referred to our hospital for aggressive management. We decided to remove the hematoma. After surgery the symptoms disappeared, and no pathological traces remained.

At followup that was continued for three months, the patient did not have any more problems and all of the neurological sequels were reversed.

## 2. Discussion

PDPH is the most common complication of lumbar puncture (LP) and manifested by signs and symptoms of excessive cerebrospinal fluid (CSF) leakage in 70% of the cases. It is thought to be caused by a downward displacement of intracranial structures that causes cerebral hypotension and stretches the intracranial pain- sensitive structures [5]. About 90% of typical PDPH may occur within the first 72 hours and 66% within the first 48 hours of LP and usually subsides in a few days with bed rest and analgesia [6].

The primary mechanism for cranial SDH after spinal anesthesia is the same as PDPH with some minor differences. Intracranial hypotension allows caudal shift of the brain, with traction on the arachnoid mater and dural veins. This causes lesions to the blood vessels and could result in blood extravasations and formation of subdural hematomas [7].

Although the true incidence of cerebral SDH after LP is unknown because not all cases are reported and probably treated without investigation [7], it is clear that the incidence of cerebral SDH is negligible in comparison with PDPH apparently. Based on a case series with a total of 1.5 million patients, hemorrhagic complications following spinal anesthesia occurred in 1 : 220,000 [8].

The changing characteristic of PDPH (including symptoms of nonpostural headaches along with focal neurological abnormalities), decreasing level of consciousness, ptosis, paresis, plegia, vomiting, headache lasting more than 5 days, [9], and unresponsiveness of the headache to ordinary treatments are warning signs and should arouse suspicion to search for a cerebral lesion [10, 11]. In case one, the headache lasted 13 days and neurologic signs were not added till the diagnosis was complete, and in case two, the signs and symptoms were severe and persistent at first and did not respond to routine treatments.

Zeidan et al. [12] state that cerebral SDH, like PDPH, grows due to leakage of CSF from the hole created by spinal needle, so the size of the needle and degree of dural tear have positive relation to PDPH and cerebral SDH. In the case of using a large needle diameter and making more attempts at inserting the needle, loss of CSF may be over 200 mL per day [4]. In both cases the 25-gauge needle was used (the smallest needle in our province), and CSF flow was detected at first attempt.

Amorim et al. [13] and Zeidan et al. [12] reviewed 35 and 25 cases of cerebral SDH following spinal anesthesia, respectively, and concluded that pregnancy, dehydration, multiple dural punctures, large dural hole, use of anticoagulants, cerebral vascular abnormalities, and brain atrophy are the risk factors. Other possible risk factors that are apparently unrelated to spinal anesthesia but may coexist in patients undergoing SDH included head trauma, coagulopathy, cerebral aneurysm, arteriovenous malformation, tumors, cerebral hypotension, chronic alcoholism, cardiovascular disease, and diabetes mellitus.

Only one of these possible risk factors was present in case one (she was pregnant). Susceptibility of pregnant patients to postdural puncture cerebral SDH might be attributed to the frequent use of epidural analgesia during labor, [14] which was absent in our cases. A review of the literature demonstrated that 20 out of 22 cases [13, 15] of cerebral SDH after inadvertent dural puncture during epidural block were obstetric patients. Some authors believe that due to haemostatic imbalance, differences in elasticity of the dura and possibly gender-based differences in cranial morphology cerebral SDH may take place more frequently in pregnant patients in comparison with other patients [16, 17]. Furthermore, there are two reports of cerebral subdural hematomas in parturient women, despite a lack of evidence of dural puncture [18, 19]. In case two, cerebral SDH occurred even in the absence of any risk factors.

Zeidan and Baraka reported that cerebral SDH after spinal anesthesia occurred more frequently on the left side [14]. In both of our cases, cerebral SDH occurred on the left side too. We did not find any propensity for left side formation of cerebral SDH on the left side in the existing literature.

The treatment of subdural hematoma may be surgical or conservative [20, 21]. Conservative treatments are recommended for small size hematomas, minimal to mild neurological manifestations, and nondeviation of cerebral structures from midline. This approach requires a close neurological and neuroradiological followup to recognize a potential worsening as early as possible [8]. Surgical decompression is considered the treatment of choice for marked and progressive neurological deficits. Our two cases had a thick hematoma, and surgical management was deemed appropriate, thus, both were treated surgically.

## 3. Conclusion

 Cerebral SDH as a serious complication of spinal anesthesia must be kept in mind and may mimic PDPH. History of LP, prolonged and nonpostural PDPH, and development of neurological symptoms should be regarded as a warning sign of an intracranial hematoma and prompt immediate diagnosis and treatment.
